# Contrasting Light Spectra Constrain the Macro and Microstructures of Scleractinian Corals

**DOI:** 10.1371/journal.pone.0105863

**Published:** 2014-08-29

**Authors:** Rui J. M. Rocha, Ana M. B. Silva, M. Helena Vaz Fernandes, Igor C. S. Cruz, Rui Rosa, Ricardo Calado

**Affiliations:** 1 Departamento de Biologia & CESAM, Universidade de Aveiro, Campus Universitário de Santiago, Aveiro, Portugal; 2 Departamento de Engenharia dos Materiais e Cerâmica/CICECO, Universidade de Aveiro, Aveiro, Portugal; 3 Laboratório de Ecologia Marinha - Instituto de Biologia Roberto Alcântara Gomes - Universidade do Estado do Rio de Janeiro, Rio de Janeiro, Rio de Janeiro, Brasil; 4 Laboratório Marítimo da Guia, Centro de Oceanografia, Faculdade de Ciências da Universidade de Lisboa, Cascais, Portugal; National University of Singapore, United States of America

## Abstract

The morphological plasticity of scleractinian corals can be influenced by numerous factors in their natural environment. However, it is difficult to identify *in situ* the relative influence of a single biotic or abiotic factor, due to potential interactions between them. Light is considered as a major factor affecting coral skeleton morphology, due to their symbiotic relation with photosynthetic zooxanthellae. Nonetheless, most studies addressing the importance of light on coral morphological plasticity have focused on photosynthetically active radiation (PAR) intensity, with the effect of light spectra remaining largely unknown. The present study evaluated how different light spectra affect the skeleton macro- and microstructures in two coral species (*Acropora formosa* sensu Veron (2000) and *Stylophora pistillata*) maintained under controlled laboratory conditions. We tested the effect of three light treatments with the same PAR but with a distinct spectral emission: 1) T5 fluorescent lamps with blue emission; 2) Light Emitting Diodes (LED) with predominantly blue emission; and 3) Light Emitting Plasma (LEP) with full spectra emission. To exclude potential bias generated by genetic variability, the experiment was performed with clonal fragments for both species. After 6 months of experiment, it was possible to detect in coral fragments of both species exposed to different light spectra significant differences in morphometry (e.g., distance among corallites, corallite diameter, and theca thickness), as well as in the organization of their skeleton microstructure. The variability found in the skeleton macro- and microstructures of clonal organisms points to the potential pitfalls associated with the exclusive use of morphometry on coral taxonomy. Moreover, the identification of a single factor influencing the morphology of coral skeletons is relevant for coral aquaculture and can allow the optimization of reef restoration efforts.

## Introduction

The morphological variability of scleractinian corals is well documented in the literature, with numerous descriptions on general shifts in colonies growth shapes [1]–[5]. Several studies have also described shifts in more specific features of corals skeletons, such as in corallite structure (e.g. septal length, columellar diameter, number of septa, theca thickness) or distance between corallites [5], [6]. This remarkable variability in scleractinian corals skeleton morphology is somehow reflected in their complex taxonomy [7]. Therefore, the analysis of interpopulational, intrapopulational and intracolonial levels of variation has been advocated by researchers to support reliable taxonomic identifications [5]. In this way, it is not surprising that morphometric analyses, at distinct levels of morphologic variation, can be a useful tool for a range of disciplines, such as physiology, ecology, biology, taxonomy, or phylogeny, that may contribute to enhance our understanding on the adaptation mechanisms, gene connectivity and habitat selection of reef building corals [6].

The aragonite (CaCO_3_) macrostructures forming the skeleton of scleractinian corals are formed under a layer of organic material secreted by cells from basal ectoderm of coral polyps [8]. Aragonite crystals precipitate in a hydro-organic gel to form microstructural units, recognized as crystallites (which form the centers of calcification) and fibers (a composite of biocrystals in which organic compounds and mineral ions interact) [8]–[12]. While several models of biomineralization have been proposed in the last years, the remarkable diversity of corals has impaired the acceptance of a single model of skeletal growth [10].

The morphology of scleractinian corals can be influenced by numerous factors in their natural environment [5], [13]. Intraspecific morphological variations among scleractinian corals have been associated with genetic variability [14], [15], competition for space [16], [17], concentration of nutrients in the water [18], [19], and with the influence of a range of environmental factors, such as light [20]–[22], depth and pressure [20], water movement [23], [24] and sedimentation rates [21], [24], [25]. Nonetheless, due to potential interactions between these factors, it is difficult to identify *in situ* the relative influence of each one of them.

Due to these complex interactions, only a few experiments have so far successfully identified single parameters affecting phenotypic plasticity in scleractinian corals. The common procedures on these experiments consist in moving colonies to new environments and register morphological shifts over time [26]. This procedure is also used in experiments that aim to identify plasticity and variation among genotypes, namely by using clonal organisms to eliminate genetic variability [13], [17], [21], [27], [28]. Therefore, it becomes evident that the only way to reliably control these variables is to perform experiments *ex situ* under controlled conditions [22].

The identification of parameters that may influence skeletal macro and microstructures organization may substantially improve coral production.

Due to the symbiotic relation of several scleractinian corals with dinoflagellates of genus *Symbiodinium*, commonly termed as zooxanthellae, several studies have addressed the importance of light in coral morphology, macrostructure organization and microstructure architecture. For example, a study performed by Todd et al. [22] suggested a relationship between *Favia speciosa* and *Diploastrea heliopora* corallite morphology and light, as corallites expanded, extended and deepened under high light conditions. Another modeling study with *Galaxea fascicularis* showed that corallite width and distance among corallites decreased with the amount of incident light, while corallite height increased with the amount of light [29]. These results suggest an optimization in corallite size and distribution to promote heterotrophic nutrition or zooxanthellae photosynthesis under low or high light conditions, respectively [29]. Most studies performed so far on the effects of light on coral morphology, either *in situ* or *ex situ*, have addressed the role of Photosynthetically Active Radiation (PAR) intensity. Curiously, only a few studies performed *ex situ* under artificial illumination have evidenced how contrasting light spectra with an identical PAR can significantly affect coral growth [30], [31]. Given that light spectra can condition the growth rate of corals, the protein content of their soft tissues and the photochemical performance of endosymbiotic zooxanthellae [30], the present study aimed to evaluate the effect of different light spectra (emitting the same PAR) in the skeletal morphology (at a macro- and microstructural level) of two symbiotic scleractinian coral species, *Acropora formosa* and *Stylophora pistillata*, maintained under controlled laboratory conditions.

## Materials and Methods

### Coral husbandry and fragmentation

One wild colony of *Acropora formosa* (∼200 mm in diameter) and one wild colony of *Stylophora pistillata* (∼150 mm in diameter) from Indonesia, termed from now as mother colonies, were acquired in a wholesale supplier operating in Portugal (solely the information on the country of origin was made available by the supplier [32]). Mother colonies were kept for 1 month in a 750 L tank (2 m×0.8 m×0.5 m), integrated in a 8000 L recirculating system operated with filtered (20 µm cartridge) natural seawater. The filtration system was composed of four protein skimmers (two AP−903 Deltec (Germany) and two 400–3×F5000 H&S (Germany)), with biological filtration being promoted by approximately 150 kg of live rock and 60 kg of aragonite sand (forming a deep sand bed with 10 cm depth). Water temperature was maintained by a Profilux II GHL (Germany) that controls both water heating (through titanium heaters) and cooling (through an Eco Cooler – Deltec, Germany). The filtration tank was also equipped with a calcium reactor PF-1001 Deltec (Germany). Water turnover in the tank holding the mother colonies through the filtration system was approximately 10 times the tank volume per hour (≈7500 L h^−1^). Additionally, the tank was also equipped with four circulation pumps (Turbelle Stream 6205, Tunze, Germany).

Water parameters were maintained as follows: salinity 35±0.5, temperature 26±0.5°C, TAN 0.05±0.01 mg L^−1^, NO_2_^−^–N 0.03±0.01 mg L^−1^, NO_3_^−^–N 0.1±0.1 mg L^−1^, PO_4_^3−^–P 0.01±0.001 mg L^−1^, pH 8.2±0.2, alkalinity 3.90±0.20 mEq L^−1^, Ca^2+^ 430±20 mg L^−1^, Mg^2+^ 1300±20 mg L^−1^. The illumination in the coral tank was provided by T5 fluorescent lamps (Sfiligoi Stealth 12×80W), delivering a PAR of 250±20 µmol quanta m^−2^ s^−1^ at the level of the colonies, with a photoperiod of 12 hours light. PAR values were measured with a Quantum Flux meter (Apogee MQ-200, USA) by placing a submergible sensor at the level of coral colonies.

After 1 month of acclimation, both mother colonies were fragmented using sterilized cutting pliers, producing 30 similar sized fragments (approximately 4 cm length×0.4 cm diameter for *A. formosa* and 1 cm length×0.7 cm diameter for *S. pistillata*) per mother colony. Coral fragments, produced from the terminal branches of each mother colony, were individually attached to a labeled plastic coral stand (Coral Cradle, UK) with epoxy resin (Aqua Medic GmbH, Bissendorf, Germany). Coral fragments of both species were stocked in the same tank of the mother colonies during one week, before the beginning of the experimental treatments (see below).

### Experimental design

**Figure 1:**
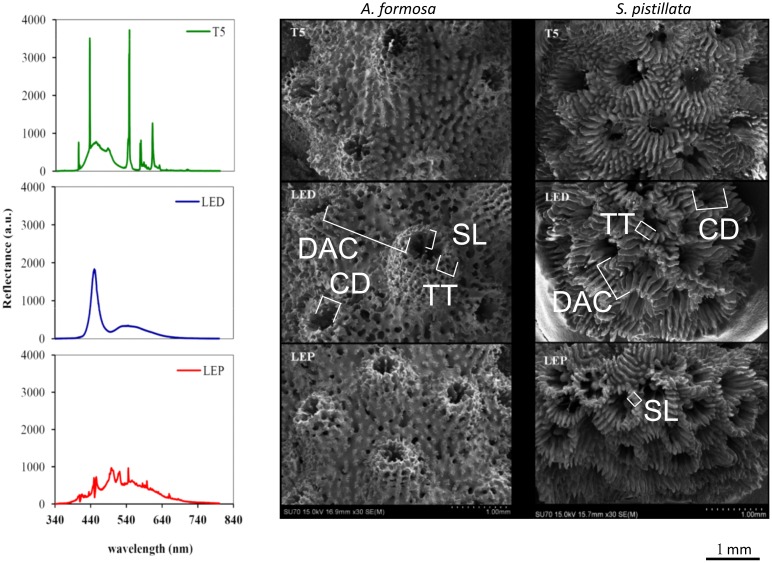
Scanning electron microphotographs (magnification: 30×). Structure of *A. formosa* radial corallites and *S. pistillata* corallites, developed under different light spectra: T5 fluorescent lamps (T5), light emitting diode (LED) and light emitting plasma (LEP). Photosynthetically active radiation (PAR) was identical to all tested light spectra: 250±20 µmol quanta m^−2^ s^−1^. Distance among corallites (DAC), corallite diameter (CD), theca thickness (TT), and septal length (SL).

Experimental treatments were performed during 6 months, using 3 different light sources with distinct spectra in the visible light wave lengths (Figure 1): 1) T5 fluorescent lamps with blue emission (T5); 2) Light Emitting Diodes (LED) with predominantly blue emission; and 3) Light Emitting Plasma (LEP) with full visible spectra emission. Reflectance spectra of lights used in the experimental treatments were measured at Ti (in the beginning of the experiment) and at Tf (in the end of the experiment) over a 340–840 nm bandwidth, with a spectral resolution of 0.33 nm, using a USB2000 spectrometer (USB2000−VIS−NIR, grating #3, Ocean Optics, USA) connected to 400 µm diameter fiberoptic (QP400−2−VIS/NIR−BX, Ocean Optics). The fiberoptic was maintained perpendicular to a reference white panel surface (WS−1−SL Spectralon Reference Standart, Ocean Optics) positioned under the light source, at a constant distance, to measure the reflected light spectra.

Light treatments were tested in 750 L experimental glass tanks, similar to the tank described above for mother colonies, with the same water flow and turnover, and connected to the same 8000 L culture system where mother colonies were stocked, in order to avoid any potential artifacts promoted by differences in water chemistry or water movement.

Each experimental tank was illuminated from above with the same PAR light intensity (250±20 µmol quanta m^−2^ s^−1^). PAR values were measured every week during the experiment with a Quantum Flux meter (Apogee MQ−200, USA) with a submergible sensor at the level of coral fragments. The distance between each light system and water surface was adjusted to have the same light PAR at the coral fragments level in all treatments. Lighting systems were operated with a photoperiod of 12 h light : 12 h dark. T5 treatment was performed employing T5 fluorescent lamps (Sfiligoi Stealth 12×80 W, Italy), mimicking the illumination employed in the tank where mother colonies were stocked. The LED treatment was performed using an 8×48 W NEPTUNE LED Reef Lighting systems (Spain), while the LEP treatment was performed under a Sfiligoi Vision Dual system, Italy (2×260W).

Twenty-seven fragments from each species were randomly selected from the initial pool of 30 fragments and distributed by the stocking tanks employed for each light spectra treatment (n = 9 for each light treatment per coral species). Coral stands were fixed on white egg-crate, to allow all coral fragments to be placed at the same water depth (≈0.3 m).

Water parameters were kept as described above for mother colonies. Partial water changes using filtered seawater (10% of total experimental system volume) were performed every other week.

### Sample preparation and porosity measurement

After 6 months of experiment the terminal branches of coral fragments were removed with sterilized cutting pliers to guarantee the utilization of coral skeleton grown after the beginning of light treatments. Fragments were identified and immersed in a 2% sodium hypochlorite solution for 12–18 h (depending on the size) to remove all the organic matter from the skeleton, and rinsed thoroughly with deionized water. After this process, the skeletons of coral fragments were dried and porosity was determined applying the “Archimedes”-method [33]. Porosity was calculated as: *x* (%) = ((w_w_−d_w_)/(w_w_−s_w_))×100, with w_w_, d_w_ and s_w_ representing the wet weight, dry weight and submerged weight, respectively.

### Sample evaluation by SEM

Samples were dried and placed on aluminum supports and covered with a conductive thin film of carbon deposition. Samples surface and morphology modification were followed by high resolution Scanning Electron Microscopy (SEM) in a HITACHI SU−70 equipped with a Bruker EDS (Energy Dispersive System) detector at an acceleration voltage of 15 keV (at RNME Pole of University of Aveiro, Portugal).

### Morphometric analyses

Morphometrics of both species were performed using the software CPCe 3.6 (Coral Point Count with Excel extensions) to analyze the images obtained with the SEM. The measurements of distance among corallites (DAC) and corallite diameter (CD, based on the mean of two greater diameters) were performed in 7 corallites of each coral fragment, for both species (n = 63; 7 corallites ×9 coral fragments per light treatment). Only top down views of corallites were considered, since features viewed at an angle can be flattened, leading to distorted measurements.

**Figure 2:**
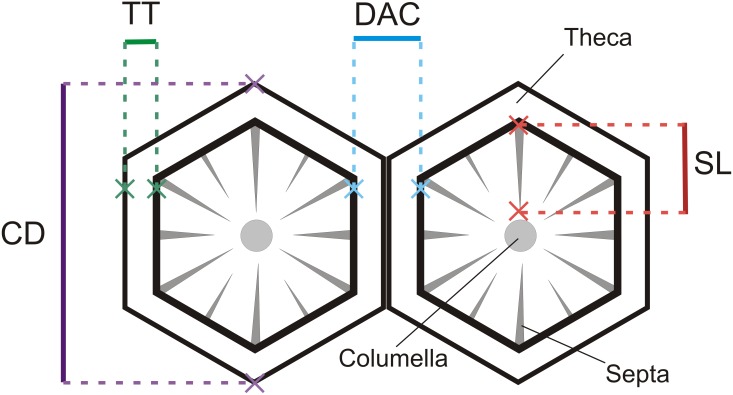
Schematic representation of corallites. Distance among corallites (DAC), corallite diameter (CD), theca thickness (TT), and septal length (SL).

In each corallite analyzed the theca thickness (TT) and the length of the septa (septal length - SL, from the intersection with the theca to the columella) were registered. For *A. formosa*, only the radial corallites were used. A schematic representation of the skeletal structures used for the morphometric analysis of both coral species is displayed in figure 2.

### Statistical analyses

Statistical analyses were performed using the software Statistica version 8.0 (StatSoft Inc.) to evaluate the existence of significant differences in the porosity (One-way ANOVA) and morphometrics (DAC, CD, TT and SL - nested ANOVA with 'fragment' as a nested variable) of coral fragment skeletons grown in the different light treatments (T5, LED and LEP, used as categorical factor) for each coral species. Assumptions of normality and homogeneity of variance were checked prior to the analysis through the Shapiro-Wilk and Levene tests, respectively. Tukey HSD post-hoc comparisons were used to determine the existence of significant differences between each species coral skeletons morphometry in the different light treatments.

Morphometric data of both species was also analyzed using principal coordinates ordination (PCO). The PCO was used to describe overall relationship among the *A. formosa* and *S. pistillata* grown in the different light treatments, respectively. The raw data matrix of morphometric data was first log (x+1) transformed, as this procedure places more emphasis on compositional differences among samples rather than on quantitative differences. After this transformation, a similarity/difference matrix was constructed using the Euclidean distance. The obtained plots (1 for each coral species) represented the distribution of specimens from the 3 light treatments according to their DAC, CD, TT and SL, together with the eigenvectors with a multiple correlation higher than 0.2. The displayed eigenvectors correspond to the obtained eigenvalues, which reflect the amount of variance explained by the PCO. Similarity percentages (SIMPER) were also explored to examine the similarity within each light treatment for each coral species. All multivariate analyses were performed using PRIMER v6 with PERMANOVA add-on (Primer-E, Ltd., Plymouth, UK).

## Results

At the end of the experiment the survival rate was 100% in all light treatments for both species. The results of porosity, imaging by scanning electron microscopy and morphometric analyses are presented below.

### Porosity

No significant differences were registered in the porosity of the skeletons of the monoclonal fragments of *A. formosa* (45.32±7.59%, 53.63±5.34% and 52.45±2.41% for T5, LED and LEP, respectively; DF = 2, F = 1.980, p = 0.2186) or *S. pistillata* (27.52±1.58%, 25.61±0.68% and 27.06±3.82%, for T5, LED and LEP respectively; DF = 2, F = 0.508, p = 0.6255) grown under the different light treatments. However, in all light treatments, the porosity of *A. formosa* skeletons was significantly higher when compared with that of *S. pistillata* (p<0.005).

### Evaluation by SEM

At the end of the experiment, *A. formosa* fragments displayed an arborescent-like growth in all light treatments, with original primary branch projecting new branches containing one axial corallite, surrounded by radial corallites. Scanning electron microphotographs (magnification 30×) of corallites from both species kept under different light treatments are presented in figure 1. We selected solely one image for each species per light treatment, as corallite patterns were similar within each light treatment for both species. Additionally we provide two supplementary figures with images of three coral fragments from each light treatment for both coral species (Figure S1 and S2 for *A. formosa* and *S. pistillata*, respectively). *A. formosa* skeletons from LED treatment evidenced corallites with larger diameter (Figure 1) and depth, which evidenced a structure with "synapticulothecate" [34] walls [35]. The costae of those radial corallites evidenced a large and defined structure running up the outside corallites wall. Corallites from skeletons of coral fragments stocked under T5 and LEP lighting presented a structure with lower size, and not as salient as corallites from coral fragments stocked under LED lighting. The costae of corallites from T5 and LEP lack presented a smaller structure mostly composed by spinules.

The corallites present in *S. pistillata* skeleton (Figure 1) evidenced a dissimilar morphology under the three light treatments tested. Corallites from LED and LEP treatments presented the costae in a vertical position, contrarily to corallites from T5 treatment whose costae was almost in a horizontal position (in the majority of corallites surveyed). The columella present in corallites from LEP treatment is close to the surface of the corallite calice, and its presence is more evident than in the corallites of fragments grown under the other light treatments.

**Figure 3:**
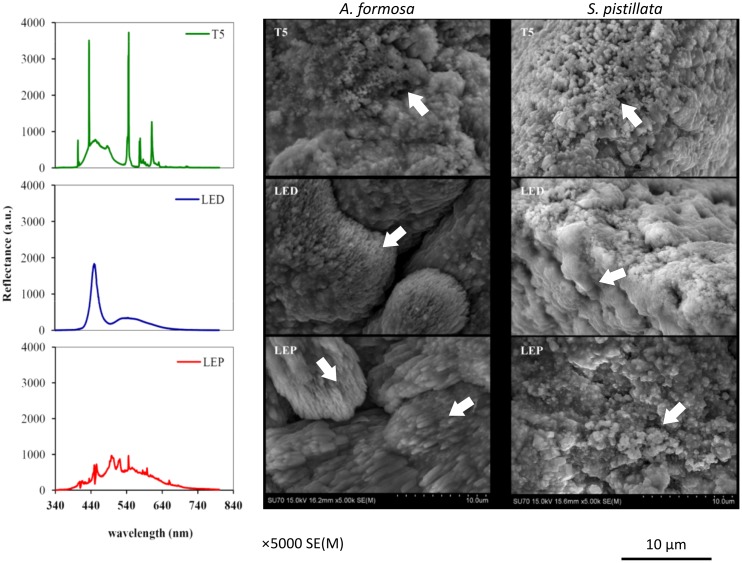
Scanning electron microphotographs (magnification: 5000×). Structure of *A. formosa* and *S. pistillata* corallites septa, developed under different light spectra: T5 fluorescent lamps (T5), light emitting diode (LED) and light emitting plasma (LEP). Photosynthetically active radiation (PAR) was identical to all tested light spectra: 250±20 µmol quanta m^−2^ s^−1^. White arrows point septal microstructures, namely crystallites form and size distribution, as well as fibers growth orientation.

The scanning electron microphotographs of corallite edge septal surface (magnification 5000×) from both species kept under the different light treatments are presented in figure 3. We selected one image for each species per light treatment, although the patterns of septal microstructures were similar inside each light treatment for both species. The form of the septa of the corallite in *A. formosa* stocked under T5 fluorescent lamps presented a microstructure mostly composed by crystallites with spherical form and homogeneous size distribution, whereas septa observed in LED and LEP treatments presented a microstructure with the presence of fibers. Those fibers observed in septa from LED treatment presented a homogeneous growth orientation in the horizontal plan and were smaller and more compact than those in the LEP treatment. Additionally, fibers observed in septa from LEP presented a growth pattern oriented to all directions in the horizontal plan.

The scanning electron microphotographs of *S. pistillata* corallite septal surface from T5 light treatment presented a distinct microstructure, composed of spherical crystallites with homogeneous size distribution. The septal microstructure of corallites from the LED treatment presented a compacted aspect, where the spherical configuration of crystallites is not evidenced. LEP septa microstructure evidenced crystallites with a larger size, when compared with those from the T5 light treatment.

### Morphometric analyses

**Figure 4:**
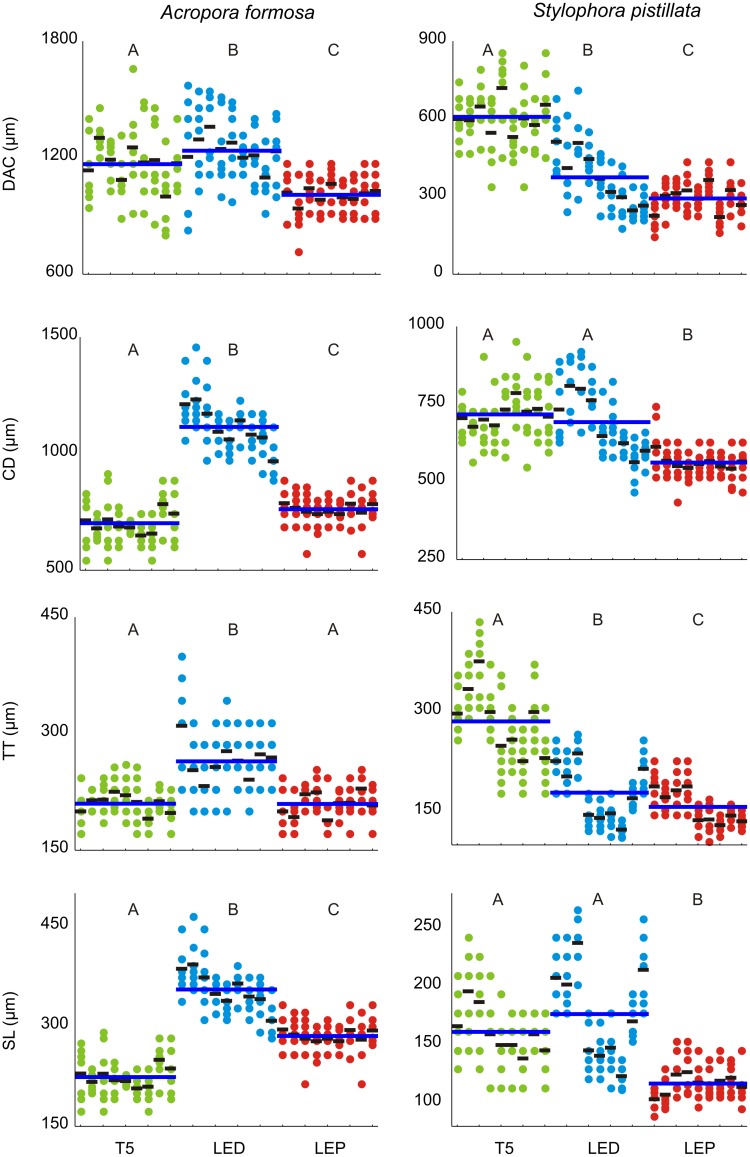
Morphometric parameters of *A. formosa* and *S. pistillata* corallites. Skeletal macrostructures obtained after SEM image analyses of coral fragments stocked under T5 fluorescent lamps (T5), light emitting diode (LED) and light emitting plasma (LEP). The blue horizontal line in each light treatment represents the average value for the distance among corallites (DAC), corallite diameter (CD), theca thickness (TT), and septal length (SL). The shorter black lines represent average measurements within each coral fragment. Different capital letters on the same graphic represent significant differences (p<0.05).

Distance among corallites (DAC), corallite diameter (CD), theca thickness (TT), and septal length (SL) registered in coral skeleton fragments from both species in the 3 light treatments are presented in figure 4.

The mean distance among corallites (± SD, for all results presented) in *A. formosa* was significantly different in all light treatments (DF = 2, F = 35.03, p<0.05), with higher values registered in the LED treatment (1236±184 µm) followed by values obtained in the T5 treatment (1167±180 µm) and in the LEP treatment (1009±101 µm). The corallite diameter (DF = 2, F = 450.90, p<0.001) and length of septa (DF = 2, F = 352.31, p<0.001) were also significantly different in all light treatments, for all comparisons. *A. formosa* fragments stocked under LED lighting presented the highest mean value of corallite diameter (1115±118 µm), followed by fragments from LEP (762±67 µm) and T5 (702±90 µm). As for corallite diameter, the highest septal length mean value was registered for corals from LED treatment (355±37 µm), followed by fragments from LEP (286±.25 µm) and T5 (224±29 µm). The theca thickness mean value was significantly higher (DF = 2, F = 71.25, p<0.01) for corals from LED treatment (264±44 µm), when compared with those from T5 (210±23 µm) and LEP (210±24 µm) treatments.

The mean distance among corallites (± SD, for all results presented) measured in *S. pistillata*, were significantly different in all light treatments for all comparisons (DF = 2, F = 207.319, p<0.001), with higher values registered in coral fragments from T5 (608±119 µm), followed by coral fragments from LED (374±128 µm) and LEP treatments (293±69 µm). The mean corallite diameter in *S. pistillata* fragments from LEP treatment (562±53 µm) was significantly lower (DF = 2, F = 80.69, p<0.01) when compared with values obtained in T5 (717±86 µm) and LEP (692±112 µm) treatments.

Theca thickness mean values were statistically different in all light treatments (DF = 2, F = 303.812, p<0.01), with the highest mean value being registered in coral fragments from the T5 treatment (285±63 µm), followed by those grown under LED (178±45 µm) and LEP (157±30 µm).

As for corallite diameter, the length of septa in *S. pistillata* fragments from LEP treatment (117±16 µm) was significantly lower (DF = 2, F = 120.522, p<0.01) when compared with values obtained in LED (167±43 µm) and T5 (161±32 µm) treatments.

**Figure 5:**
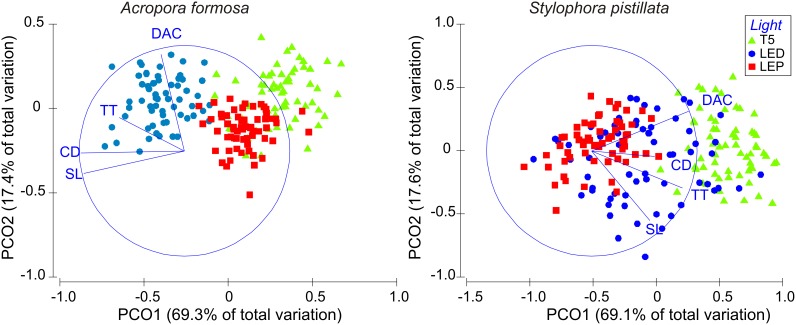
Principal component ordination based on *A. formosa* and *S. pistillata* morphometry. Distance among corallites (DAC), corallite diameter (CD), theca thickness (TT), and septal length (SL) of coral skeletons from fragments stocked under T5 fluorescent lamps (T5), light emitting diode (LED) and light emitting plasma (LEP). Eigen vectors of multiple correlations (>0.2) are represented.

Figure 5 shows a principal component ordination (PCO) based on morphometric characteristics of the coral species studied. The first two axes of *A. formosa* PCO represent approximately 87% of total variation. Both ordinations evidenced the differences in morphometric parameters between light treatments. The horizontal axis of variation separated specimens stocked under LED light treatment, with the corallite diameter and septal length more strongly influencing this pattern of distribution. The vertical axis maximized the differences between skeletons from LED, T5 and LEP, mainly due to distance among corallites.

The first two axes of the PCO for *S. pistillata* represent approximately 87% of the total variation recorded. The corallite diameter contributed for the differentiation between corals stocked under T5 light from those stocked LED and LEP, while theca thickness and the distance among corallites contributed to maximize the differences between skeletons from LED, T5 and LEP.

## Discussion

The results of the present study provide a new insight into how light spectra can affect the macro and microstructure of the skeletons displayed by scleractinian corals. The experimental procedure allowed the study of a single factor (light spectra), once the interactions with Photosynthetic Active Radiation (PAR), water parameters, and genetic variability were excluded, though: 1) the application of the same PAR intensity in all light spectra treatments, 2) the utilization of the same water with a common life support system for all treatments, 3) the utilization of the same equipment (circulation pumps) in all experimental tanks, 4) the equivalent position of coral fragments and pumps in the tanks, and finally 5) the utilization of monoclonal fragments as experimental replicates for both species (a procedure that greatly reduced genetic variability) [13], [17], [21], [27]. Therefore, it becomes evident that the spectral emission of light should receive a renewed attention by the scientific community studying the effects of light on zooxanthellate corals, in order to complement data provided by studies addressing the effect of PAR.

Understanding the light requirements of corals, especially for those species being cultured, is fundamental to achieve optimal production. The growth of scleractinian corals can be influenced by three physiological processes: 1) photosynthesis, 2) heterotrophic feeding, and 3) calcification [36]. The use of artificial illumination emitting in different wavelengths of visible light, but with the same PAR, has already been shown to affect coral growth [30], [31]. In this topic, it is already documented the importance of blue light to the photosynthetic performance of zooxanthellae [37]–[39]. It has been suggested that higher calcification rates in scleractinian corals could be strongly related with autotrophy and endosymbionts activity [40].

The effect of light in scleractinian corals is widely described in literature. Studies on morphology suggest that corals might undergo plastic depending on the surrounding environment. A study performed by Todd et al. [22] with *Favia speciosa* and *Diploastrea heliopora* suggests a relationship between corallite morphology and light, detecting that corallites expand, extend and deepen under high light conditions. Another study performed by Crabbe and Smith [29] with *Galaxea fascicularis* showed that corallite width and distance among corallites decreased with the amount of incident light, while corallite height increased with the amount of light. The increase of corallite depth with increasing light can be related with a strategy of achieving optimal internal irradiances for the photosynthetic activity of dinoflagellates harbored within coral tissues [41].

In the present study different light spectra, with the same PAR intensity, promoted differences detectable by corallite morphometry, namely a significantly higher distance among corallites, corallite diameter, theca thickness and septal length on *A. formosa* and *S. pistillat*a fragments grown under the LED blue spectra, when compared to the fragments grown under the LEP full visible spectra. It is well known that the amount of energy in light depends on the frequency of the wavelength. Blue light has a higher frequency than red light for example, and a photon of blue light has more energy than a photon of red light [42]. Consequently it is expected that in spite of the utilization of the same PAR, blue light treatments such as LED could provide more energy than LEP. Therefore, we can hypothesize that the differences in morphometric parameters evaluated for both species in the LED treatment, as well as for some parameters (e.g. DAC or TT) in the T5 treatment (which contain a higher percentage of emission in blue spectra than LEP), can be promoted by corals as a way to optimize internal radiances for their endosymbiotic zooxanthellae.

As already referred, the morphometric analysis of corallite structures have been used to support coral taxonomy in the last decades. According to Veron [43], while this method is objective, numerically rigorous, and repeatable, differences between corallites evidenced by morphometrics can be readily detected by skilled observers. This methodology can present several limitations related with spatial variation within the same colony (e.g. old corallites near the base of mature *Pocillopora damicornis* can be more similar with basal corallites of other *Pocillopora* species than with the peripheral corallites of their own colony) [43].

The general microstructure of the coral skeleton has been established for many years [8]; however, the arrangement of fibbers and centers of calcification can result in a wide variety of tri-dimensional microstructural patterns, and no single model available so far is satisfactory to describe coral skeletogenesis [10], [12].

The use of skeletal morphology complemented with information resulting from molecular approaches is a powerful tool for coral taxonomy [44], [45]. Skeletal microstructure has been linked to molecular phylogenetic techniques [14] to partially support phylogenetic relationships based on microstructural patterns. Nonetheless, the exact microstructural patterns for scleractinian corals remain uncertain [46]. As recognized by Veron in a recent overview on coral taxonomy [43], environment-correlated microskeletal variation in hard corals continues to be largely overlooked, even at higher taxonomic levels, although such variations can be easily observed in most member species of families Faviidae and Mussidae.

According to Schmidt-Roach et al [47] fine-scale morphological variation is useful to differentiate clades, and provides an excellent signature of the evolutionary relationships among genetic lineages. Still, taxonomic decisions based on morphometric measurements, accounts for differences related to environmental factors between habitats and for within-colony variability [48]. The differences in skeletal microstructure of coral fragments originating from the same mother colony, promoted by different light spectra, can contribute for morphological investigations on the two studied coral species.

A study published by Rocha et al. [30] showed that blue light spectra from LED promoted higher specific growth rates (mean ± SD) in *A. formosa* (0.0031±0.0005% day^−1^) when compared with coral fragments grown under T5 (0.0019±0.0004% day^−1^) and LEP (0.0011±0.0004% day^−1^) lights. Blue light spectra also positively affected the specific growth rates registered for *S. pistillata*, since the coral fragments grown under the full light spectra of LEP presented significantly lower values of specific growth rate (0.0014±0.0003% day^−1^) when compared with coral fragments grown under T5 (0.0022±0.0006% day^−1^) and LED (0.0023±0.0003% day^−1^). While these differences may somehow help to explain the differences in microstructure, we cannot claim that they are indeed correlated. Moreover, as no significant differences in porosity were detected, any further discussion on this topic would be too speculative.

The possibility to shape the skeleton structure of cultured corals can also contribute to the optimization of reef restoration efforts [49], [50]. By manipulating certain factors *ex situ*, such as light color simulating light extinction with ocean depth, or light intensity, one can promote the development of skeleton structures that may enable corals to thrive better once they are transplanted to their new natural environment.

Overall, results from the present experiment evidence the key role played by light color, resulting from the emission wave length, in both the coral skeleton macro- and microstructure. It is shown that experimentation *ex situ* under controlled conditions and relying on monoclonal coral fragments can open a new window of opportunity to evaluate individual parameters affecting the skeleton structure of zooxanthellate scleractinian corals.

## Supporting Information

Figure S1 **Scanning electron microphotographs (magnification: 30×).** *Acropora formosa* radial corallites of coral skeletons from fragments stocked under different light spectra: T5 fluorescent lamps (T5), light emitting diode (LED) and light emitting plasma (LEP). Photosynthetically active radiation (PAR) was identical to all tested light spectra: 250±20 µmol quanta m^−2^ s^−1^.
(TIF)

Figure S2 **Scanning electron microphotographs (magnification of 30×).** *Stylophora pistillata* corallites of coral skeletons from fragments stocked under different light spectra: T5 fluorescent lamps (T5), light emitting diode (LED) and light emitting plasma (LEP). Photosynthetically active radiation (PAR) was identical to all tested light spectra: 250±20 µmol quanta m^−2^ s^−1^.
(TIF)
